# Synthesis of well–dispersed silver nanorods of different aspect ratios and their antimicrobial properties against gram positive and negative bacterial strains

**DOI:** 10.1186/1477-3155-11-42

**Published:** 2013-12-20

**Authors:** Animesh K Ojha, Stefan Forster, Sumeet Kumar, Siddharth Vats, Sangeeta Negi, Ingo Fischer

**Affiliations:** 1 Motilal Nehru National Institute of Technology Allahabad, Allahabd 211004, India; 2 Institute of Physical and Theoretical Chemistry, University of Würzburg, Am Hubland, Wuerzburg D-97074, Germany

**Keywords:** Silver, Nano rods, TEM, Antimicrobial activities

## Abstract

In the present contribution, we describe the synthesis of highly dispersed silver nanorods (NRs) of different aspect ratios using a chemical route. The shape and size of the synthesized NRs were characterized by Transmission Electron Microscopy (TEM) and UV-visible spectroscopy. Longitudinal and transverse absorptions bands confirm the rod type structure. The experimentally recorded UV-visible spectra of NRs solutions were fitted by using an expression of the extinction coefficient for rod like nano structures under the dipole approximation. Simulated and experimentally observed UV-visible spectra were compared to determine the aspect ratios (R) of NRs. The average values of R for NR1, NR2 and NR3 solutions are estimated to be 3*.*0 ± 0*.*1, 1*.*8 ± 0*.*1 and 1*.*2 ± 0*.*1, respectively. These values are in good agreement with those obtained by TEM micrographs. The silver NRs of known aspect ratios are used to study antimicrobial activities against *B. subtilis (gram positive)* and *E. coli (gram negative)* microbes*.* We observed that the NRs of intermediate aspect ratio (R = 1.8) have greater antimicrobial effect against both, *B. subtilis (gram positive)* and *E. coli (gram negative).* The NRs of aspect ratio, R = 3.0 has better antimicrobial activities against gram positive than on the gram negative.

## Introduction

Almost fifty years ago Richard Feynman suggested that the properties of materials can be modified by miniaturization and atomic level control [1]. In this regard, significant progress has been made by the scientific community to produce reproducible nanometer-sized inorganic structures including spheres [1,2], wires [3], and rods [3]. The unique, physical, chemical, and biological properties of nanometer-sized materials have recently attracted a great deal of interest in the scientific community. Size and shape dependent behavior of physical and chemical properties of materials have led scientists to design and develop different methods for synthesizing the nanomaterials. Among the physical and chemical properties that systematically depend on the variation in shape and size are light absorption, magnetic, and electrical characteristics. Nanomaterials have been used in applications such as; sensors, catalysis, electronics, surface enhanced Raman spectroscopy, and diagnostic imaging [1,4,5]. Well defined silver nanorods and nanowires are desirable for their applications in different fields. Silver nanorods and nanowires have been prepared using different methods [6-12].

The antibiotic resistance among microbes is one of the biggest challenges which have given a blow to all possible human medicine and drug system. Thus it has become urgent and highest priority to discover new medicines and new drugs with better efficacy and target specificity. Nanoparticles being unique in their physical and chemical properties have interesting effects on living matters and are considered as effective and potent tools in the medicinal chemistry. Nanoparticles can be different shapes and morphology, and nanorod is one of them with most of the research focused on metal made nanoparticles [13]. They have an aspect ratio ranging from 1–20. The aspect ratio is one of the most important parameter of nanorods which controls their chemical, physical and biological properties [14]. Antimicrobial effects of silver metal ions and silver salts are well understood. In this manuscript, we address the question how different silver nano particle sizes, as represented by their aspect ratio, affect the antimicrobial properties of nanostructure. This issue is not yet understood.

In a study by Fayaz et al. [15], biosynthesis of highly stable AgNPs from silver nitrate solution is reported using fungus Trichoderma viride. The synthesized nanoparticles had been characterised by different techniques. The antibacterial activities of antibiotics such as ampicillin, kanamycin, erythromycin, and chloramphenicol in presence of Ag nanoparticles were investigated against gram positive and negative bacteria. The antibacterial activities of these antibiotics were found to be increased in the presence of AgNPs against test strains. The study revealed that the combination of antibiotics with AgNPs have better antimicrobial effects. Guzman et al. [16] had synthesized Ag nanoparticles via chemical route and studied their antimicrobial activities against Escherichia coli, Pseudomonas aeruginosa, and Staphylococcus aureus bacteria as function of nanoparticle concentration and size. They observed a strong antimicrobial activity for a concentration of silver nanoparticles less than 7 ppm. In a recent work by Taglietti et al. [17], the mechanism of antimicrobial activities of silver nanoparticle coated with glutathione (GSH) were investigated on gram positive and gram negative bacterial strain in two ways (i) by dispersing the silver nanoparticles in solution (ii) grafted on thiolfunctionlized glass surfaces. The antimicrobial activities of GSH coated nanoparticles dispersed in solution were found to be more intense compared to the nanopartcile grafted on thiolfunctionlized glass surfaces due the penetration of Ag^+^ of the colloid into the cytoplasm of E-coli bacteria.

At nanoscale, it is the size and shape only which affects the overall properties. Here, a new scheme is applied for synthesizing well dispersed silver nanorods of different aspect ratios using chemical method. The scheme is an extension of a method proposed by Sau et al. [2] for the synthesis of silver nanorods. The size and shape of the synthesized nanorods are characterized by UV-visible spectroscopy and TEM. The silver nanorods of known aspect ratio were further used for studying the antimicrobial effects against *B. subtilis* and *E. coli* microbes*.*

## Experimental details

### Synthesis of silver nanorods

Silver nitrate (AgNO_3_), sodium borohydride (NaBH_4_), ascorbic acid (C_6_H_8_O_6_), sodium hydroxide (NaOH) and sodium citrate of analytical grade were purchased from Sigma Aldrich and used without further purification. A colloidal silver seed solution was prepared in deionized water based on the method described by Creighton et al. [18]. However, several aspects were modified compared to the original procedure.

First of all 2 ml of a 2.5 mM solution of AgNO_3_ and 2 ml of a 2.5 mM solution of citrate were mixed and 0.6 ml of a 10 mM solution of NaOH were added. Then deionized water was added until a total volume of 20 ml resulted. The solution was vigorously stirred until a slight clouding disappeared. Then 0.6 ml of a 10 mM solution of ice cold (0°C) of NaBH_4_ was added while stirring. After 30 s the stirring was stopped. This product constituted the seed solution that was used for the further synthesis of nanorods.

**Figure 1:**
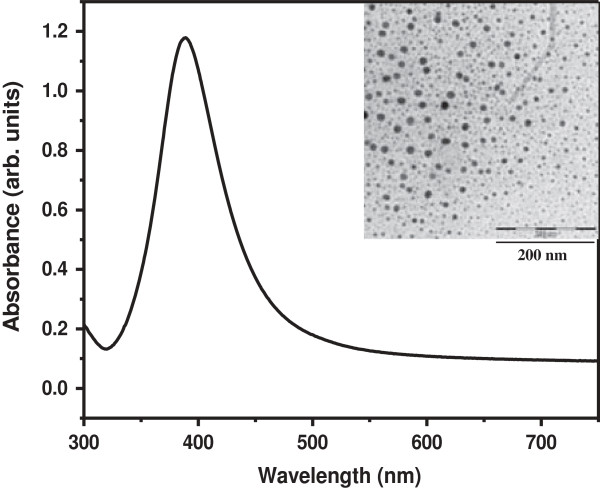
UV-visible spectrum of synthesized silver nano seeds and its TEM micrograph.

The mean size as well as the size distribution of the particles in the silver seed solution was determined by performing UV–Vis absorption and TEM measurements as shown in Figure 1. The average size of synthesized Ag seed was calculated using TEM micrographs and its value was found to be 7 ± 2 nm. It was also confirmed by the position of absorption peak which mainly corresponds to the seed type of structure (see Figure 1). For synthesizing Ag nanorods of three different aspect ratios, three stock solutions of 10 mM AgNO_3_, 100 mM ascorbic acid, and a 80 mM solution of the surfactant Cetyl trimethylammonium bromide (CTAB) were prepared separately as reported in earlier study [19]. The CTAB solution was prepared by adding deionized water to CTAB and shaking it until it dissolved. Note that diluting the CTAB solution down to 20 mM did not change the results described below appreciably. Using the stock solutions, we prepared three sets of solutions containing 0.25 ml of AgNO_3_, 0.50 ml of 100 mM ascorbic acid, and 10 ml of saturated CTAB solution and mixed them properly. Thereafter, 1.0 ml, 0.5 ml, and 0.25 ml of synthesized seed solution was added to set one, two and three, respectively and at the end, 0.10 ml of 1 M NaOH solution was also added to each set. In order to mix all the added components uniformly, the reaction solution was shaken gently. After 10 minutes, a change in color took place in all the three sets of solution. The color of the solutions depends on the seed concentrations added in the final solution. A different color of each nanorod solutions indicates the formation of nanorods of different aspect ratios in the solutions. In order to separate the nanorods from spheres and surfactant, 5 ml solution of each nanorod solution was centrifuged at 6000 rpm for 30 mins. After centrifuging, the surfactant was removed and the precipitate thus obtained was further re-dispersed in deionized water. The re-dispersed solutions were used for UV-visible spectroscopy, TEM and antimicrobial measurements. These three sets of solutions are labelled as NR1, NR2 and NR3 and correspond to the addition of 1.0 ml, 0.5 ml and 0.25 ml of seed solution, respectively.

### UV-visible and TEM measurements

UV–visible spectra of the three sets of re-dispersed silver nanorod and seed solutions were measured in a UV-visible spectrometer from JASCO in the spectral range 300–800 nm. Samples for transmission electron microscopy (TEM) were deposited onto 300 mesh copper TEM grids coated with 50 nm carbon films. Samples were suspended in water and thus, directly added drop-wise to the grid. The excess water was allowed to evaporate in air. The grids were examined with a JEOL 2010 microscope with ultra-high resolution (UHR) microscope using a LaB6 filament operated at 200 KeV.

### Characterizations of silver nanorods by TEM and UV-visible spectroscopy

**Figure 2:**
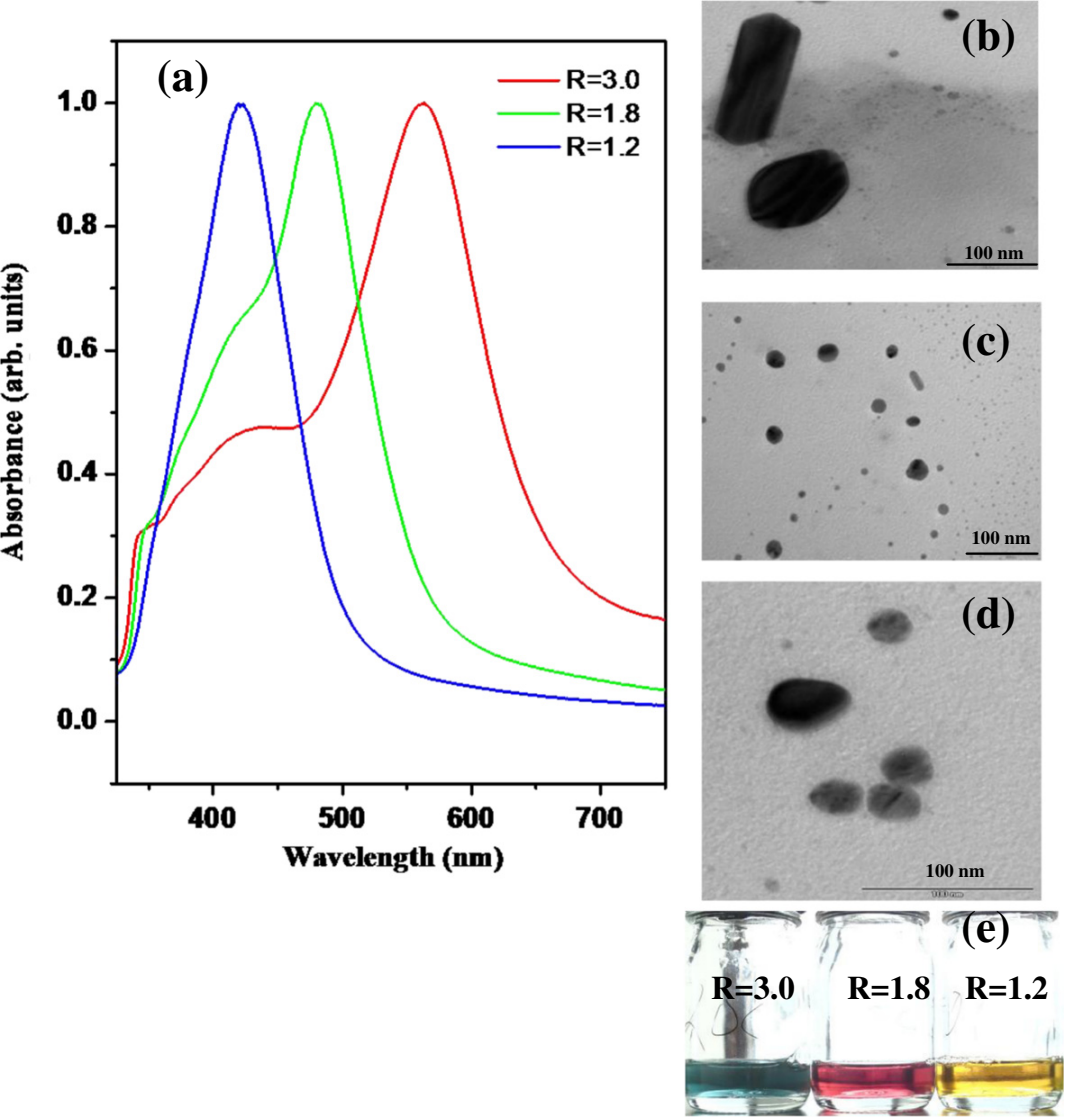
UV-visible spectrum of synthesized silver nano nanrods solutions, NR1, NR2, and NR3 of different aspect ratios (a) TEM micrograph of NR1 (b) TEM micrograph of NR2 (c) TEM micrograph of NR3 (d) and a picture of synthesized nanorods solutions (e).

The UV-visible spectra of three nanorod solutions NR1 (red line), NR2 (green line) and NR3 (blue line) recorded for the spectral range 300–800 nm are shown in Figure 2 (a). The UV-visible spectra of each nanorod solution exhibit two absorption bands of different intensity, corresponding to the longitudinal and transversal surface plasmon modes, abbreviated “longitudinal band” and “transverse band”. In general, the more intense longitudinal band appears due to the contribution from the dipole oscillation along the long axis of the nanorods, while the less intense transverse band corresponds to a dipole oscillation along the short axis of the nanorods. The colloidal solutions of silver nanorods have also been characterized using TEM. The TEM micrographs of nanorod solutions NR1, NR2 and NR3 are shown in Figure 2 (b), (c) and (d), respectively. In order to show how the absorption spectrum of the nanorod solutions depends on aspect ratios of the rods, pictures of the color of the synthesized solutions NR1, NR2 and NR3 were taken and are shown in Figure 2 (e). TEM images of all our samples (NR1– NR3) exhibit formation of well-dispersed NRs in colloidal solutions. The different mean size of the three solutions becomes evident in the TEM images. Under the quasistatic approximation, the aspect ratio of a NR and positions of absorption maxima in UV-visible spectrum can be correlated to the extinction coefficient C_*ext*_ by the following relationship [20]:

(1) $$\frac{C_{\mathrm{ext}}}{\mathrm{NV}}=\frac{2\pi {\epsilon^{\frac{3}{2}}}_{m}}{3\lambda }\;\sum_{j=A}^{C}\frac{1/{P_{j}}^{2}\;\epsilon_{2}\left(\omega \right)}{{\left[\epsilon_{1}\left(\omega \right)\;+\frac{1-P_{j}}{P_{j}}\;{\epsilon_{m}}^{2}\right]}^{2}+{\epsilon_{2}}^{2}\left(\omega \right)}$$

where N, V, ϵ_m_ and λ are the number of particle per unit volume, the particle volume itself, the medium dielectric constant and the wavelength of the incident light, respectively. The geometrical factor P_j_ provides information about the depolarization along the three axes A, B and C. For rods, the effect of depolarization along B and C axes is equal and corresponds to the short axis, described by the particle diameter (d), while the A axis represents long axis, described by the particle length (L). The values of P_A,_ P_B_ and P_c_ are given as:

(2) $$P_{A}=\frac{1-e^{2}}{e^{2}}\left[\frac{1}{2e}\ln\left(\frac{1+e}{1-e}\right)-1\right]$$

(3) $$P_{A}=P_{B}=\frac{1-P_{A}}{2}$$

where

(4) $$e=\sqrt{1-{\left(\frac{d}{L}\right)}^{2}}$$

The ratio L / d is called aspect ratio of the nanorods. The total absorption of the nanorods is calculated by summation over the three axes A, B and C along which polarization oscillation can take place. It is quite obvious from Eq. (1) that the mean absorption frequencies for NRs depend on their structure (aspect ratio), but also on the dielectric constant of the surrounding medium [20]. In order to fit the experimentally observed absorption spectra using Eq. (1), the frequency dependent values of *ϵ*_1_ and *ϵ*_2_ have been taken from Ref [21]. Since the NRs solutions are re-dispersed in water, we have taken *ϵ*_m_ = 1*.*77 (dielectric constant of water at visible wavelengths). Neglecting the scattering of light by the nanostructures and comparing the maxima of the simulated absorption spectrum (using equation (1)) with the observed peak positions in their optical absorption spectra (Figure 2 (a)), the average aspect ratio R of NRs for NR1, NR2 and NR3 are estimated to be 3*.*0 ± 0*.*1, 1*.*8 ± 0*.*1 and 1*.*1 ± 0*.*1, respectively. From the TEM micrographs, the mean value of aspect ratios R were determined to be of 3.0, 1.8 and 1.2 for NR1, NR2 and NR3, respectively. Thus, the R-values obtained from the UV-visible spectra and from the TEM micrographs are in excellent agreement. It is important to note that the variation of aspect ratios of nanorods in the NRs solution depends on the seed concentrations added to the solution. The added concentrations of seed solution form the basis for nanorods of different aspect ratios. The nanorods of desired aspect ratio may be synthesized by adding appropriate volume of seed solution. In the forthcoming section the antimicrobial activity of silver nanorods of known aspect ratios against *B. subtilis* and *E. coli* microbes will be discussed.

### Strains and diseases associated with *B. subtilis* and *E. coli* microbes

For the investigation of the particle’s antimicrobial activity, strains of *B. subtilis* and *E. coli* microbes were used. They were isolated from river Ganges flowing through the city of Allahabad, India. *B. subtilis*, a gram positive bacterium, produces a toxin called subtilisin. Subtilisin can cause allergic reactions if there is repeated exposure in high concentrations and has been associated with pulmonary and bronchial diseases [22]. Shiga toxin producing *E. coli* (STEC) causes serious systemic toxemic complications and is considered as food and water borne disease [23]. *E. coli* is also responsible for causing infections in human and animals when water or food contaminated with feces is consumed. *E. coli* can get into cooked food and meat during processing and is the most common way of infection among developed countries. Some strains of *E. coli* like Enterohemorrhagic *E. coli* (EHEC) can cause anemia or kidney failure and more commonly urinary infection [24].

### Preparation of microbial culture and plates for test

A microbial culture of 10^6^ Colony Forming Units (CFU) was freshly prepared from stock solution for analysis. Microbes were inoculated in sterilized and autoclaved Muller Hinton Broth (MHB) to the conditions mentioned on the box. 15 gm of MHB was dissolved in 1000 ml flask containing 500 ml of distilled water, pH 7.0 and incubated for 24 hours at 28 ± 2°C in an incubator shaker at 120 rpm. Plates were prepared of Muller Hinton Agar (MHA) to check the antimicrobial activity of filter paper disc coated with silver solution of nanorods with different aspect ratios. Discs were 8 mm in size and prepared from sterilized filter paper.

### Assay for antimicrobial activity of silver nanorods of different aspect ratios against B. subtilis and E. coli microbes

Modified Bauer-Kirby disc diffusion method in was followed to study antimicrobial activity of silver nanorods particle [14]. Discs used were made up of sterilized filter paper and had a diameter of 8 mm. These discs were then impregnated with 0, 20, 30 50 μl silver NRs solution of concentration 1 × 10^-6^ M and were placed onto Muller Hinton Agar (MHA) plates made up of autoclaved MHA media and had bacteria swabbed (100 μl). These plates were then incubated overnight at 28 ± 2°C and the zone of inhibition around the discs was measured. Large zones of inhibition around the disc indicated susceptibility of microbe toward that aspect ratio nanorod while small zones or no zones of inhibition indicated resistive microbes.

### Determination of minimum inhibitory and bactericidal concentration

Minimum inhibitory concentrations (MIC) and Minimum bactericidal concentration (MBC) of silver NRs based on their aspect ratios has been studied and it is found that the NRs with aspect ratio, 1.8 have high antimicrobial activity. The method followed was according to the Clinical Laboratory standards Institute (CLSI) recommended standards. 50 μl of respective bacterial culture suspension with 10^6^ CFU/ml (approximate) was taken and inoculated into 1 × 10^-6^ M concentration of silver nanorods. Tetracycline was used as standard.

## Results and discussion

### Antimicrobial activity

**Figure 3:**
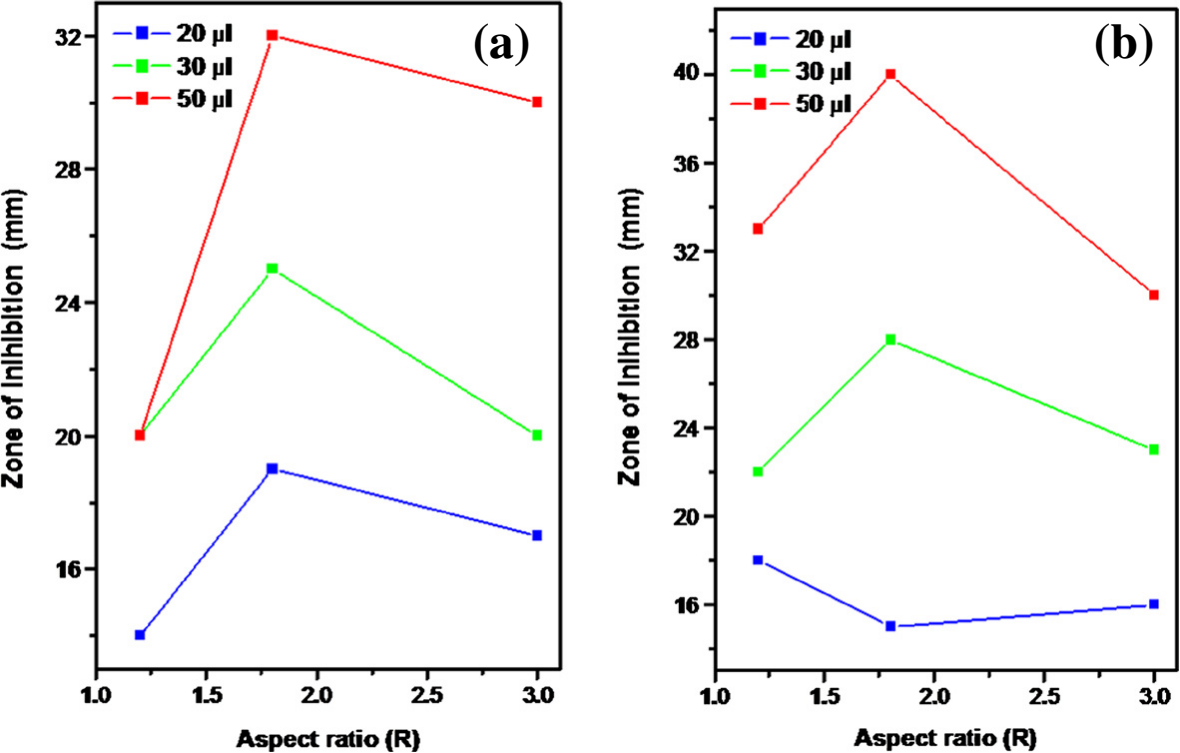
**Variation of inhibition zones as function of aspect ratios of nanorods (a) *E. coli *** **(gram-ve) (b) *B. subtilis *** **(gram + ve) microbes.**

**Figure 4:**
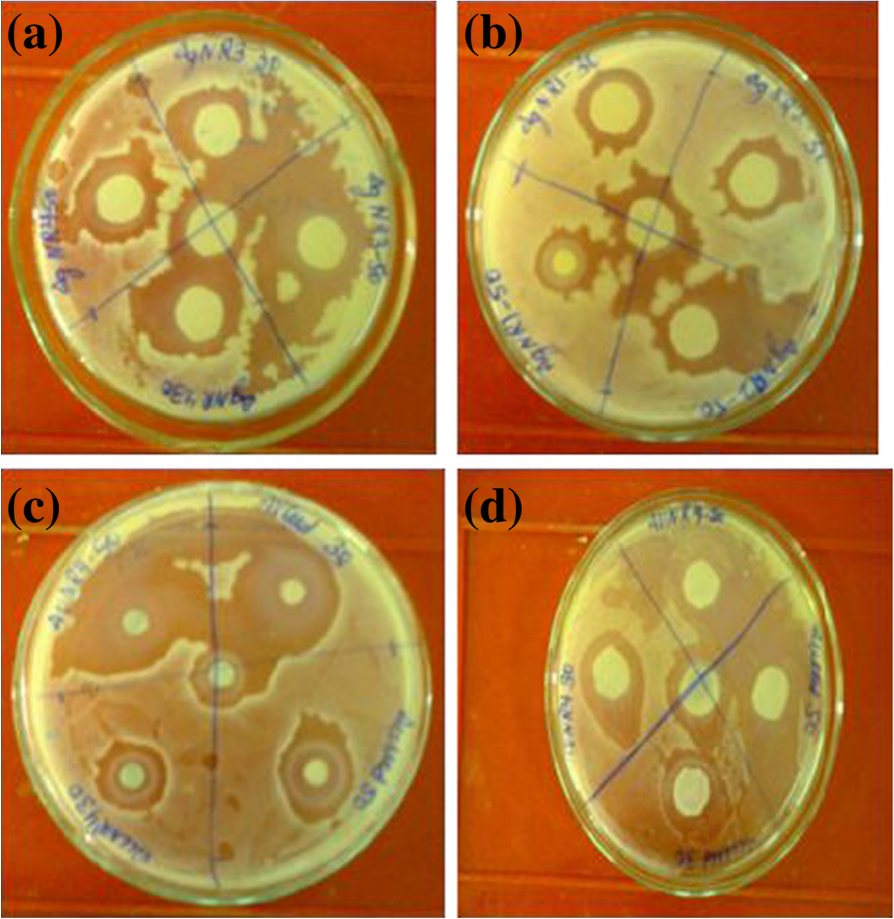
**Pictures of antimicrobial activity of (a) NR1 at 30 μl against *B. subtilis *** **(gram + ve) (b) NR2 at 30 μl against *E. coli *** **(gram-ve) (c) NR1 at 30 μl against *B. subtilis *** **(gram + ve) *E. coli *** **(gram-ve) (d) Silver nano seeds against *E. coli *** **(gram-ve).**

Nanoparticles have high surface to volume ratio and reactive facets. Their nano-size is responsible for generating reactive oxygen species for the damage to the microbial or cell membrane lipids and for the damage to the DNA which ultimately leads to death of the microbes or cell [22-25]. The zone of inhibition as function of aspect ratio R at different volume of NRs solution for *B. subtilis (gram + ve)* and *E. coli (gram-ve)* is shown in Figure 3 (a) and (b), respectively. The antimicrobial activities of NR1 (R = 3.0), NR2 (R = 1.8) and NR3 (R = 1.2) against *B. subtilis (gram + ve)* and *E. coli (gram-ve)* at 30 μl volume of NRs solution are shown in Figure 4 (a-d). The antimicrobial activity of NRs of R = 1.2 at concentration 30 μl and 50 μl on gram positive bacteria is shown in Figure 4 (a) while Figure 4 (b) shows the activity NRs of R = 3.0 against gram negative bacteria. Figure 4 (c) shows the antimicrobial activity against gram negative bacteria and Figure 4 (d) shows the effect of the silver seed solution against *E. coli*. The inhibition zones for different NRs solution at different volumes are presented in Table 1.

**Table 1:** **The measured values of inhibitions zone at different concentration of silver nanorods (NR1, NR2 and NR3) against *B. subtilis (gram + ve) and E. coli *** **(gram-ve)**

**S. no.**	**Aspect ratio**	***E. coli (gram-ve)***				***B. subtilis (gram + ve)***			
	**(R)**	**100 Microliters of solution used**				**100 Microliters of solution used**			
		**Singular zone of inhibitions (mm) at different volume of nanorods solutions**				**Singular zone of inhibitions (mm) at different volume of nanorods solutions**			
		**0 μl**	**20 μl**	**30 μl**	**50 μl**	**0 μl**	**20 μl**	**30 μl**	**50 μl**
1	1.2	0	14	20	20	0	18	22	33
2	1.8	0	19	25	32	0	15	28	40
3	3.0	0	17	20	30	0	16	23	30

From the test results it may be concluded that silver particles with intermediate aspect ratio (R = 1.8) has high antimicrobial activity against both gram positive and gram negative microbes and it has been observed that the silver nanorods are more effective against gram positive strains compared to gram negative. Silver NRs with the lowest aspect ratio (R = 1.2) have same antimicrobial effect with 30 and 50 μl against gram negative bacteria however in case of gram positive bacteria the antimicrobial effect has been increased significantly upon increasing the volume by 30 to 50 μl. Thus, for R = 1.2 the diameter of inhibition zone is directly proportional to the volume of the NRs solution for positive bacterial strain. The NRs of aspect ratio R = 1.2 have better results on gram positive than that of gram negative microbes. The nanorods of aspect ratio (R = 1.8) have 19 mm zone of inhibition on use of 20 μl of the NRs solution for gram negative microbes. Further while changing the NRs volume from 20 to 50 μl, the diameter of inhibition zone is increased to 13 nm for gram negative and 25 nm for gram positive microbes. In this case, we see a continuous increase of zone of inhibition, proportional to the volume size. By looking at the data presented in Table 1, we may conclude that NR2, (R = 1.8) has better zone of inhibition against both gram positive and negative microbes. However, the NRs of aspect ratio (R = 3.0) are found to have relatively better antimicrobial activities for gram positive than that of gram negative microbes. The results obtained against both the microbes confirm that NRs of aspect ratio (R = 1.8) has good antimicrobial activity. The different strength of antimicrobial activities of silver nanorods towards gram positive and negatives may be due to the different cell structure (one with cell wall, the other one without), different metabolic rate and activity, different physiology. They also interact differently with the nanorods. It may be due to the fact that gram positive bacteria have a large number of free amines and carboxyl groups on their surfaces, while gram negative ones have the ability to protect themself from the antimicrobial agents**.** Microbes break down the antibiotics by defensive enzymes system before it disrupts the cell wall. To make more effective medicines or drugs, it is possible and required to use the conjugated effect of silver nanostructures and antibiotics tailored to selectively interact with molecules of interest. Once silver nanostructures get inside the cell, they can interfere with DNA replication. The inhibition zone is a function of Ag^+^ concentration, shape and size of the nanoparticles [15-17]. In the present case, the decrease of singular zone of inhibition upon increasing the aspect ratio from 1.8 to 3.0 for both the microbes may be due to the effect of shape of the nanorods. The nanorods of aspect ratio 3.0 may not be releasing Ag^+^ easily from the nanorods due to the strong confinement leading to less interaction of Ag^+^ with cells of both the microbes. As a consequence, the size of inhibition zone decreases. In author’s opinion, this is the first report which says that a specific aspect ratio is required for getting maximum zone of inhibition for gram positive and negative microbes.

## Conclusions

The silver nanorods of different aspect ratios were synthesized through chemical routes. The synthesized nanorods were characterized by UV-visible spectroscopy and TEM measurements. Each nanorods solution exhibits two absorption bands corresponding to the longitudinal and transverse modes of surface plasmon. The values of aspect ratios calculated using UV-visible spectra were found to be in good agreement with those obtained from TEM micrographs. The NRs solutions of know aspect ratio were directly used for investigating their antimicrobial activities against *B. subtilis (gram + ve)* and *E. coli (gram-ve)* and *microbes* by taking different volumes of the NR solutions. It was found that the NR2 solution with aspect ratio 1.8 have the largest antimicrobial effects against both the microbes. Further, we also observed that by increasing the volume of the NR solutions, the diameter of the inhibition zones also increased for each NR solutions.

## Competing interests

The authors declare that they have no competing interests.

## Authors’ contributions

AKO, SF, and SK carried out the synthesis, characterizations, and drafting of the manuscript. IF supervised the synthesis and characterization of the nanorods and contributed to the drafting of the manuscript. The SV and SN carried out the antimicrobial measurements of synthesized nanorods of known aspect ratios over gram positive and negative microbes. All authors read and approved the final manuscript.
